# Scaling of maneuvering performance in baleen whales: larger whales outperform expectations

**DOI:** 10.1242/jeb.243224

**Published:** 2022-03-02

**Authors:** Paolo S. Segre, William T. Gough, Edward A. Roualdes, David E. Cade, Max F. Czapanskiy, James Fahlbusch, Shirel R. Kahane-Rapport, William K. Oestreich, Lars Bejder, K. C. Bierlich, Julia A. Burrows, John Calambokidis, Ellen M. Chenoweth, Jacopo di Clemente, John W. Durban, Holly Fearnbach, Frank E. Fish, Ari S. Friedlaender, Peter Hegelund, David W. Johnston, Douglas P. Nowacek, Machiel G. Oudejans, Gwenith S. Penry, Jean Potvin, Malene Simon, Andrew Stanworth, Janice M. Straley, Andrew Szabo, Simone K. A. Videsen, Fleur Visser, Caroline R. Weir, David N. Wiley, Jeremy A. Goldbogen

**Affiliations:** 1 Hopkins Marine Station, Stanford University, Pacific Grove, CA 93950, USA; 2 Department of Mathematics and Statistics, California State University, Chico, Chico, CA 95929, USA; 3 Institute of Marine Sciences, University of California, Santa Cruz, Santa Cruz, CA 95064, USA; 4 Cascadia Research Collective, Olympia, WA 98501, USA; 5 Department of Biological Science, California State University, Fullerton, Fullerton, CA 92834, USA; 6 Marine Mammal Research Program, Hawaii Institute of Marine Biology, University of Hawaii at Manoa, Kaneohe, HI 96744, USA; 7 Zoophysiology, Department of Bioscience, Aarhus University, 8000 Aarhus C, Denmark; 8 Division of Marine Science and Conservation, Duke University Marine Laboratory, Beaufort, NC 28516, USA; 9 Marine Mammal Institute, Hatfield Marine Science Center, Oregon State University, Newport, OR 97365, USA; 10 Stanford University, Stanford, CA 94305, USA; 11 University of Alaska Fairbanks, Fairbanks, AK 99775, USA; 12 Department of Natural Sciences, University of Alaska Southeast, AK 99835, USA; 13 Marine Mammal Research, Department of Ecoscience, Aarhus University, 8000 Aarhus C, Denmark; 14 Department of Biology, University of Copenhagen, 2200 Copenhagen N, Denmark; 15 Department of Biology, University of Southern Denmark, 5230 Odense M, Denmark; 16 Southall Environmental Associates, Inc., Aptos, CA 95003, USA; 17 SR3, SeaLife Response, Rehabilitation and Research, Des Moines, WA 98198, USA; 18 Department of Biology, West Chester University, PA 19383, USA; 19 Greenland Climate Research Centre, Greenland Institute of Natural Resources, Nuuk 3900, Greenland; 20 Nicholas School of the Environment and Pratt School of Engineering, Duke University Marine Lab, Beaufort, NC 28516, USA; 21 Kelp Marine Research, 1624 CJ Hoorn, The Netherlands; 22 Institute for Coastal and Marine Research, Nelson Mandela University, Gqeberha 6031, South Africa; 23 Department of Physics, Saint Louis University, St Louis, MO 63103, USA; 24 Falklands Conservation, Stanley F1QQ 1ZZ, Falkland Islands; 25 Alaska Whale Foundation, Petersburg, AK 99833, USA; 26 Department of Freshwater and Marine Ecology, IBED, University of Amsterdam, 1090 GE Amsterdam, The Netherlands; 27 Department of Coastal Systems, Royal Netherlands Institute for Sea Research, Texel, 1790 AB Den Burg, The Netherlands; 28 NOAA/Stellwagen Bank National Marine Sanctuary, Scituate, MA 02066, USA

**Keywords:** Maneuverability, Agility, Scaling, Cetacean, Swimming

## Abstract

Despite their enormous size, whales make their living as voracious predators. To catch their much smaller, more maneuverable prey, they have developed several unique locomotor strategies that require high energetic input, high mechanical power output and a surprising degree of agility. To better understand how body size affects maneuverability at the largest scale, we used bio-logging data, aerial photogrammetry and a high-throughput approach to quantify the maneuvering performance of seven species of free-swimming baleen whale. We found that as body size increases, absolute maneuvering performance decreases: larger whales use lower accelerations and perform slower pitch-changes, rolls and turns than smaller species. We also found that baleen whales exhibit positive allometry of maneuvering performance: relative to their body size, larger whales use higher accelerations, and perform faster pitch-changes, rolls and certain types of turns than smaller species. However, not all maneuvers were impacted by body size in the same way, and we found that larger whales behaviorally adjust for their decreased agility by using turns that they can perform more effectively. The positive allometry of maneuvering performance suggests that large whales have compensated for their increased body size by evolving more effective control surfaces and by preferentially selecting maneuvers that play to their strengths.

## Abstract (precis)

Maneuvering performance decreases with body size in large whale species, but larger whales maneuver better than expected.

## INTRODUCTION

For many animals, the ability to maneuver is a critical aspect of survival. Maneuverability, broadly defined as the ability to change speed and direction (Dudley, 2002), plays an important role in behaviors such as competition, courtship, hunting and escaping predators (Altshuler, 2006; Clark, 2009; Fish et al., 2003; Walker et al., 2005; Wilson et al., 2015). Whether in terrestrial, aerial or aquatic media, the ability to maneuver makes life in a three-dimensional environment possible. However, because of its complex and voluntary nature, the role that maneuverability plays in shaping the higher-level processes of ecology and evolution remains poorly understood. Is maneuverability more or less influential for determining a species' evolutionary trajectory than other factors such as aerobic capacity, locomotor efficiency or behavior? Many studies have used behavioral or mechanical means to reduce the complexity of self-selected locomotion, with the aim of investigating the physiological controls and performance capabilities of stereotyped maneuvers (i.e. Clark et al., 2013; Higham et al., 2001; Iriarte-Díaz and Swartz, 2008; Jackson and Dial, 2011; Read et al., 2016; Ros et al., 2011; Socha, 2005). Benchmarking important stereotyped maneuvers, such as those used for social displays or predatory strikes, can be predictive of fitness and survival in certain contexts (Altshuler, 2006; Walker et al., 2005). However, many organisms rely on suites of complex and varied maneuvers to negotiate their three-dimensional environment and to perform a range of daily tasks. Therefore, understanding the physiological determinants and the ecological and evolutionary implications of maneuvering performance requires a more holistic approach (Dakin et al., 2018).

Quantifying maneuvering performance comes with many unique challenges (Dakin et al., 2020). When an individual selects a maneuver to use from a menu of possible options, it takes into account many different factors including motivation (Irschick, 2003), complexity (Fish et al., 2018; Segre et al., 2019), efficiency (Wilson et al., 2013) and proficiency (Dakin et al., 2018). As a result, there is a high level of variability in the maneuvers or sequences of maneuvers that are performed. One way to deal with this variability is to study maximal performance (Garland, 1985). However, maximal performance can only be measured if the individual has a high level of motivation, which is difficult to determine for wild animals (Irschick et al., 2005). After all, even in a life-or-death situation, why would an animal expend excess energy to escape from a predator that it knows it can easily outmaneuver with submaximal performance? Furthermore, a high motivational state may prevent an animal from using the breadth of its maneuvering capabilities, causing it to use only a subset of maneuvers that it can perform well (Segre et al., 2015). For example, humans can run backwards, but spinning around and running forwards is more effective for outrunning a competitor (Angelino et al., 2021). In this case, the runner substitutes a difficult simple maneuver with a complex sequence of easier and more effective maneuvers. A high-stakes situation does not guarantee a high level of maneuvering performance or the use of a wide range of maneuvers.

A second way to deal with the inherent variability of maneuvering performance is to measure a large number of voluntary maneuvers, performed across a range of behavioral circumstances (Segre et al., 2015). If enough maneuvers of a given type are detected, the maneuvering performance metrics follow identifiable sample distributions. The distributions' central tendencies correlate with maximum performance and capture the intrinsic differences between individuals (Dakin et al., 2020; Segre et al., 2015). Meanwhile, the types of maneuvers individuals use reflect their behavioral preferences. This method has limitations: by itself it cannot accurately assess maximal performance, and it only works for measuring commonly used maneuvers. This recently developed approach has been used with captive hummingbirds to elucidate the physiological drivers of individual maneuvering ability (Segre et al., 2015), the effects of environmental conditions on maneuvering performance (Segre et al., 2016a) and how flight agility evolves (Dakin et al., 2018). A similar approach has also been used to compare maneuvering performance across species of dragonfly (Bomphrey et al., 2016). This method requires repeated sampling of known individuals over extended periods of time, high-resolution data on body position and orientation, and high-throughput analyses. To date, this has only been possible in captive animals. In this study, we used bio-logging data and a computational approach to quantify the maneuvering performance of free-swimming baleen whales and to answer the question: how does maneuverability scale with body size in the world's largest animals?

It is difficult to overstate the important effect that body size has on physiological processes (Schmidt-Nielsen, 1975). As body dimensions increase, surface area increases with length squared, and volume (and therefore mass) increases with length cubed. Because of this basic geometric property, large animals do not look, function or behave like small animals. Scaling affects physiological functions as varied as metabolism (Kleiber, 1947; Nagy, 2005), body structure (Kahane-Rapport and Goldbogen, 2018) and locomotion (Alexander, 2005; Vogel, 2008), which influence higher-level processes such as ecology, evolution and behavior (Dakin et al., 2018; Dial et al., 2008; Domenici, 2001; Goldbogen et al., 2019). Because of the difficulties associated with quantifying maneuverability, the effects of body size on maneuvering performance remain poorly understood. Generally speaking, the maneuverability of running, flying and swimming animals decreases with increasing body size (Cloyed et al., 2021). In aquatic organisms, the ability to accelerate diminishes as animals get bigger; however, the precise scaling relationships are far from conclusive and may vary across taxa (reviewed in Domenici, 2001; Vogel, 2008). Likewise, the turning performance of swimming animals decreases with body size (reviewed in Fish et al., 2018), but the specifics defy simple interpretation. Different taxa use different methods of turning (e.g. sharks, Hoffmann et al., 2019; rays, Parson et al., 2011; sea lions, Fish et al., 2003; zebrafish, Danos and Lauder, 2007; and humpback whales, Edel and Winn, 1978), and performance is contingent on both the shape of the control surfaces involved (Woodward et al., 2006) and the flexibility used to reorient the body (Kajiura et al., 2003; Porter et al., 2009; Segre et al., 2019). Even less is known about how pitch changes and rolls scale with body size, although the control of these maneuvers is also highly dependent on the shape of the lift-generating surfaces (Weber et al., 2014; Woodward et al., 2006) and the locomotor strategies involved. In aquatic organisms, maximum speed might hold some clues for how maneuvering performance scales with body size. Maximum swimming speed is related to burst power (Segre et al., 2020), which may influence the performance of certain maneuvers (demonstrated in other taxa; Altshuler, 2006; Segre et al., 2015). There is some indication that maximum speed plateaus with body size (Cloyed et al., 2021; though this is not fully resolved), suggesting that larger animals have less mass-specific power available for maneuvering (Segre et al., 2020, their eqn 10). However, although power-generating capability can help to overcome inefficient morphology, it is not the only factor that determines maneuvering performance (Dakin et al., 2018). In all of these analyses, there is a notable lack of data from the biggest of organisms. By focusing on the maneuvering performance of whales, we can directly compare large animals that have similar body shapes, control surface types and maneuvering strategies (Woodward et al., 2006). Despite their enormous size, whales make their living as voracious predators (Goldbogen et al., 2019). To catch their much smaller, more maneuverable prey, they have developed several unique locomotor strategies that require high energetic input, high mechanical power output and a surprising degree of agility (Goldbogen et al., 2017a). Yet, it is precisely their ability to catch large quantities of smaller prey that has shaped their physiology, size and behavior, and has allowed them to become successful predators with a worldwide distribution and a keystone role in supporting ocean ecosystems (Goldbogen et al., 2019; Roman et al., 2014; Slater et al., 2017).

**Fig. 1.**
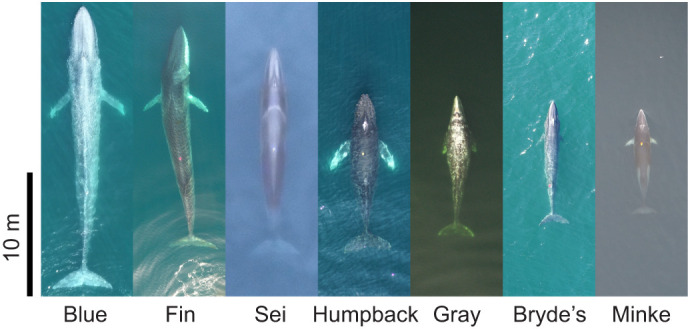
Aerial photographs of a blue, fin, sei, humpback, gray, Bryde's and minke whale.

To better understand the ecology and evolutionary biology of baleen whales, we ask the question: how does maneuverability scale with body size? We hypothesize that absolute maneuvering performance decreases with increasing body size (H1). In other words, we predict that smaller whales accelerate, roll and change direction faster than larger whales. Baleen whales span an immense range of body sizes (Kahane-Rapport and Goldbogen, 2018), from the smallest minke whales (5 m, 2000 kg) to the largest blue whales (30 m, 185,000 kg), and we expect that smaller individuals are more maneuverable than larger individuals. This result would not be surprising; however, understanding the baseline relationship between body size and absolute maneuvering performance sets the stage for investigating more nuanced hypotheses about scaling. Specifically, we hypothesize that maneuvering performance scales allometrically with body size (H2). Maneuvering performance is constrained by the differential scaling of muscle force-generating capabilities, surface area of the flippers and flukes, and body dimensions (Fish, 2002, 2004; Vogel, 2008; Webb and De Buffrénil, 1990). We can use these relationships to create predictions for how accelerations, rolls and direction changes should scale with body mass (*m*), under the assumption of isometry. Particularly, we expect accelerations and turns to scale with *m*^–1/3^ and rolling performance to scale with *m*^–2/3^ (as derived in the Materials and Methods). However, in baleen whales, many morphological properties scale allometrically, meaning that whales of different sizes have different body shapes (Fig. 1). Whether these allometric scaling relationships cause larger whales to overperform or underperform the maneuvering capabilities predicted by their body size remains an open question. If this was the case, we would expect the scaling coefficients to differ from the values predicted by isometry (*m*^–1/3^ and *m*^–2/3^). We also hypothesize that large whales compensate for any limitations on their maneuverability by preferentially using maneuvers that they can perform more effectively (H3). Blue whales execute high-speed turns by banking their body inwards so that they can use lift from their extended flippers and their substantial dorso-ventral flexibility to turn faster (Segre et al., 2019). By substituting a simple maneuver that they do not perform well (laterally flexing their body) with a complex sequence of maneuvers that they are adept at performing (rolling inward, pitching-up to take advantage of their dorso-ventral flexibility, rolling back to upright), blue whales behaviorally overcome the limitations of their morphology to increase their turning performance (Segre et al., 2019). We predict that this pattern will extend across species and may depend on body size. Specifically, we want to know whether larger whales use behavioral compensation to overcome morphological constraints and increase their maneuverability.

Finally, we expect the maneuvering performance of large whales to exhibit high variability. Variability caused by morphological differences (both within and across species) may lead to a better understanding of how specific maneuvers are controlled and evolved (Dakin et al., 2018). The generalized rorqual body plan is sleek and streamlined, with high-aspect-ratio flippers and flukes (Woodward, 2006). However, humpback whales and the more distantly related gray whales have more ellipsoidal bodies and control surfaces with dramatically different shapes. Therefore, we expect humpback whales to perform faster rolls (owing to their much longer flippers) and slower accelerations (owing to their stouter body shape) than other rorqual species (Edel and Winn, 1978; Fish and Battle, 1995; Woodward et al., 2006). Other sources of variation may be confounding, such as individual variability in behavior. Rorqual whales will often enter behavioral states where they spend long periods of time focusing on a single task, such as migrating, sleeping, raising juveniles or feeding. This is one of the challenges of using the measure-of-center approach to quantify performance in free-living individuals: short-duration, high-resolution tag deployments only capture a snapshot of their life and this may be problematic in animals that spatially and temporally segregate their behaviors. Although the primary purpose of this study is to understand how maneuverability scales with body size in the largest of animals, we also examine some of the explanatory and confounding factors behind the patterns in the data.

## MATERIALS AND METHODS

### Bio-logger data collection

Between 2010 and 2020, we deployed multi-sensor bio-loggers on minke whales (*Balaenoptera bonaerensis* Burmeister 1867; *N*=20; West Antarctic Peninsula), inshore Bryde's whales (*Balaenoptera edeni* Anderson 1879; *N*=6; South Africa), gray whales (*Eschrichtius robustus* Lilljeborg 1861; *N*=5; Washington), humpback whales (*Megaptera novaeangliae* Borowski 1781; *N*=131; various worldwide locations), sei whales (*Balaenoptera borealis* Lesson 1828; *N*=2; Falkland Islands), fin whales [*Balaenoptera physalus* (Linnaeus 1758); *N*=31; Azores, California and Greenland] and blue whales [*Balaenoptera musculus* (Linnaeus 1758); *N*=85; California and Azores]. Over the course of the study, we used three types of bio-logging tags equipped with three-axis accelerometers, three-axis magnetometers and temperature-calibrated depth sensors. DTAGs (v2 and v3; Johnson and Tyack, 2003) were deployed on humpback, fin and blue whales; Acousonde tags (Greeneridge Sciences; Burgess, 2009) were deployed on humpback and blue whales; and CATS tags (Customized Animal Tracking Solutions; Goldbogen et al., 2017b) were deployed on all seven species. Sampling frequencies of the instruments varied depending on the tag specifications, but were all above 10 Hz and were decimated in post-processing to 5, 10 or 25 Hz. Additionally, swimming speed was calculated using either flow noise captured by the hydrophones (DTAG and Acousonde) or the background vibrations captured by the high-frequency accelerometers (CATS, using the un-decimated data), and calibrated using the orientation-corrected depth rate method (Cade et al., 2018; Goldbogen et al., 2006). In total, we deployed tags on 280 individuals for a combined 4037 h of recorded data (Table 1).

**Table 1.**
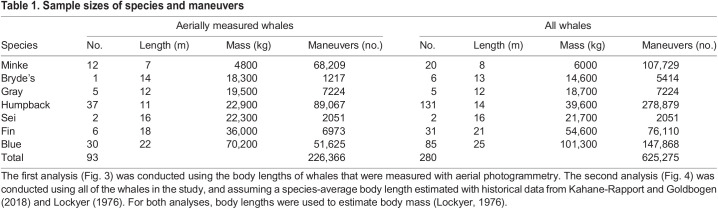
Sample sizes of species and maneuvers

Data collection was conducted under permits from the United States National Marine Fisheries Services (16111, 14809, 15271, 19116, 14809, 21476, 14122, 18059, 23095, 19091, 18529, ACA2015-011 and NMS MULTI-2017-007), the Falkland Islands Government (R11.2017 and R23.2018), the South African Department of Forestry, Fisheries, and the Environment (RES 2018/63 2019/57), the Direção Regional dos Assuntos do Mar, Secretaria Regional do Mar, Ciência e Technologia of the Azores, and the Greenland Government. Procedures were approved by all of the relevant institutional animal care and ethics committees.

### Aerial photogrammetry and morphological measurements

Whenever possible, we used remotely operated unoccupied aircraft systems (UASs) to collect aerial images of tagged whales. Over the decade of the data collection for this project, there have been rapid advancements in UAS technology, and therefore we used many different models with varying capabilities (DJI Phantom 4, DJI Phantom 4P, DJI Inspire 2, FreeFly Alta 6, LemHex-44, Aerial Imaging Solutions APO-42). UASs were equipped with either laser or barometric altimeters. When using the less accurate barometric altimeters, we took photographs of the boats to ground-truth the altimeter readings (Burnett et al., 2018; Durban et al., 2016). We used the altimeter readings and camera parameters to obtain the scale of each photograph. The distance (m) from one corner of the photograph to the diagonally opposite corner is represented by the equation:
(1)

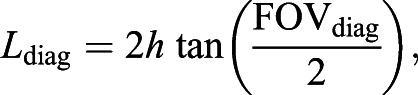
where *h* is the altimeter height (m) and FOV_diag_ is the diagonal field of view of the camera (rad). This relationship was used to convert measurements of the distance from rostrum to tail notch of each whale, from pixels to meters. Details about the UASs, the piloting methods and the accuracy of measurements can be found in Table S1 and in previously published papers (Durban et al., 2015, 2016; Gough et al., 2019; Torres and Bierlich, 2020). We measured the body lengths of 93 individual whales from all seven species (Table 1).

### Identification of maneuvers

To calculate the body orientation, the accelerometers and magnetometer were first aligned with the body axis of the whale (Johnson and Tyack, 2003), and then filtered with a low-pass Butterworth filter designed to remove the fluctuations caused by the fluking motion (forward–backward, cut-off frequency 0.08 Hz, Python3 SciPy implementation, Segre et al., 2019; species-average stroke frequencies range from 0.19 to 0.37 Hz, Gough et al., 2019). The aligned and filtered accelerometers and magnetometer data were used to calculate the globally referenced pitch, roll and heading of the whale (Cade et al., 2021; Johnson and Tyack, 2003). The speed and depth data were smoothed using a low-pass Butterworth filter designed to remove fluctuations caused by sampling error (forward–backward, cut-off frequency 0.4 Hz; Python3 SciPy implementation; Segre et al., 2019, 2020).

**Fig. 2.**
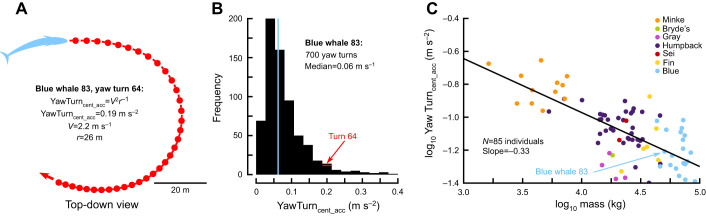
**Pure-yaw turning performance decreases with body size in baleen whales.** (A) A blue whale performs a single yawing turn to the right. *V*, velocity; *r*, radius. (B) Over the course of a 29 h tag deployment, the same blue whale performed 700 pure-yaw turns, with a median centripetal acceleration of 0.06 m s^–2^. (C) The median centripetal acceleration produced during pure-yaw turns decreases with body size across seven species of large whales.

Using the body orientation and speed data, we identified six types of translational and rotational maneuvers (Table 2, Fig. S1), and from each maneuver we extracted a performance metric that can be compared across individuals and species (Fig. 2A; Segre et al., 2020). Forward accelerations were identified by searching for sections of the data where the speed increased from a local minimum to a local maximum, and where the change in speed was >0.5 m s^−1^. Accelerations at the surface are difficult to measure, owing to the tag repeatedly emerging from the water, and therefore we only included maneuvers where the minimum depth for the entire acceleration was >3 m. From each forward acceleration maneuver we measured the maximum acceleration (Acc_max_).

**Table 2.**
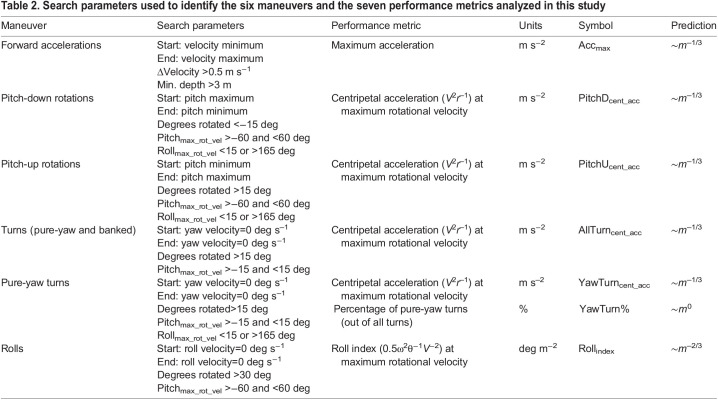
Search parameters used to identify the six maneuvers and the seven performance metrics analyzed in this study

Pitch-down rotations were defined as maneuvers where the globally referenced pitch changed from a local maximum to a local minimum, rotating more than 15 deg downward. To ensure that the whale was rotating in the dorso-ventral plane (pitching), we only included maneuvers where the whale was rolled upright (|roll| <15 deg) at the time of the maximum rotational velocity. We also included the few pitch-down rotations where the whale was upside down (|roll|>165 deg at the time of the maximum pitching velocity) and where the globally referenced pitch changed more than 15 deg upward while moving from a local minimum to a local maximum. Because roll was inaccurate at extreme pitch angles (owing to the underdefined pitch-roll-heading coordinate system; Segre et al., 2019), we only included maneuvers where the pitch angle at the time of maximum rotational velocity was between −60 and 60 deg. From each pitch-down rotation we calculated the centripetal acceleration (PitchD_cent_acc_) at the time of maximum rotational velocity.

Pitch-up rotations were defined as sections of the data where the globally referenced pitch changed from a local minimum to a local maximum, with an upward rotation of more than 15 deg. Again, we only included maneuvers where the whale was rolled upright (|roll|<15 deg) at the time of the maximum rotational velocity. We also included the few pitch-up rotations where the whale was upside down (|roll|>165 deg at the time of the maximum pitching velocity) and where the globally referenced pitch changed more than 15 deg downward while moving from a local maximum to a local minimum. In both cases, we only included maneuvers where the pitch angle was between −60 and 60 deg at the time of maximum rotational velocity. From the pitch-up maneuvers, we calculated the centripetal acceleration (PitchU_cent_acc_) at the time of maximum rotational velocity.

To identify turns, we searched for sections of the data where the globally referenced yawing velocity (analogous to a change in compass bearing; derived from the body orientation) started at zero, increased and then returned to zero, and where the excursion of the turn was more than 15 deg. To ensure that the whale was turning in a level plane, we only included maneuvers where the pitch at the time of the maximum rotational velocity was between −15 and 15 deg. These turns included both pure-yaw turns (|roll at maximum rotational velocity| <15 or >165 deg) and banked turns (roll at maximum rotational velocity >15 and <165 deg). We measured the centripetal acceleration at the time of maximum rotational velocity for all turns (AllTurn_cent_acc_) and for just the pure yaw turns (YawTurn_cent_acc_).

To identify rolls, we searched for maneuvers where the rolling velocity (derived from the body orientation) began at zero, increased and then returned to zero, and where the total roll excursion was >30 deg. Because roll was inaccurate at extreme pitch angles, we only included maneuvers where the pitch angle at the time of maximum rolling velocity was between −60 and 60 deg. At the time of maximum rolling velocity, we calculated an index of rolling performance (Roll_index_) which accounts for angular acceleration and swimming speed, and is described in detail in the next section.

Finally, the methods used for calculating swimming speed (flow noise; background accelerometer vibrations) are less accurate at lower speeds (Cade et al., 2018; Goldbogen et al., 2006). Therefore, we only included forward accelerations where the minimum swimming speed for the entire maneuver was >1 m s^−1^. Likewise, we only included pitch-changes, turns and rolls where the swimming speed at the time of maximum rotational velocity was >1 m s^−1^.

### Selection of performance metrics

For each category of maneuver, we selected a performance metric designed to: (1) reflect the physical forces required to change speed or direction; (2) benchmark the performance of maneuvers even if they have variations in shape and trajectory; and (3) compare performance across individuals and species (Fig. 2, Table 2). For forward acceleration maneuvers, the metric was simply maximum acceleration (Acc_max_ in m s^−2^). Because of Newton's second law, maximum acceleration is directly proportional to the maximum propulsive forces produced by the fluke strokes:
(2)

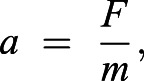
where *F* is force, *m* is mass and *a* is acceleration. For rotations such as pitch changes and turns, we measured centripetal acceleration (PitchD_cent_acc_, PitchU_cent_acc_, AllTurn_cent_acc_, YawTurn_cent_acc_, all in m s^−2^). Like forward acceleration, centripetal acceleration is proportional to the amount of force that can be directed radially inward to affect the turn, and whales use their flippers and body flexion to produce and orient the centripetal force (Segre et al., 2019). The centripetal acceleration (rad s^−2^) can be expressed in two ways:
(3)

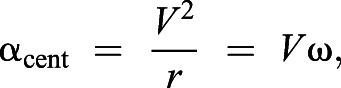
where *V* is translational velocity (or swimming speed; m s^−1^), *r* is radius (m) and ω is angular velocity (rad s^−1^). The first formulation demonstrates the trade-off between translational velocity and the radius of a turn: for a given amount of centripetal force, the whale must sacrifice speed to perform a tighter turn, or vice versa. This makes centripetal acceleration an ideal metric for comparing turns with different speeds and trajectories, in order to benchmark how much radially directed force a whale can produce. The second formulation is the method we used to calculate centripetal acceleration: by multiplying translational velocity with angular velocity at the time of the maximum angular velocity.

Finally, comparing long-axis rolls requires a different type of performance metric. Long-axis rolls performed at speed are executed by using the extended flippers to generate asymmetrical lift (i.e. one flipper angled down, the other flipper angled up; Segre et al., 2016b). The angular acceleration of a roll (α_roll_ in rad s^−2^) is influenced by the posture and the hydrodynamic properties of the flippers, the moment of inertia of the body (*I* in kg m^2^) and the translational velocity (*V* in m s^−1^):
(4)

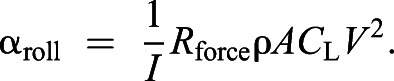


The posture of the flippers is reflected by the variable *R*_force_, which is the radius at which the lifting force is applied (in meters). This takes into account the angle of the flippers, the length of the flippers and the radius of the body. The hydrodynamic properties include the planform area of the flipper (*A* in m^2^), the coefficient of lift (*C*_L_, which accounts for the angle of attack) and the density of water (ρ). The complete derivation of this equation can be found in Segre et al. (2016b, their eqn A1) and is shown in the Supplementary Materials and Methods. Eqn 4 illustrates how angular rolling acceleration (*a*_roll_) are heavily influenced by translational velocity (*V*). Therefore, a performance metric for rolls must measure acceleration while accounting for the fact that it is much easier to roll at faster swimming speeds. The metric we selected to illustrate the ‘difficulty’ of performing a roll is:
(5)

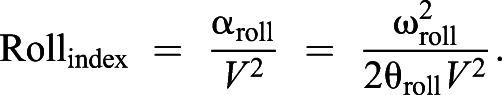


Because angular accelerations are difficult to measure and prone to propagative error (Segre et al., 2015), we chose to measure angular velocity (ω_roll_ in rad s^−1^) and the roll excursion (θ_roll_ in rad) instead. Roll_index_ (converted to deg m^−2^ for legibility) can be used to compare rolls of different angular excursions performed across a range of swimming speeds.

### Data aggregation

For each of the metrics associated with the six maneuvers, an individual's performance follows a right-skewed distribution (similar to a lognormal distribution). To benchmark the performance of individual whales, we took the median value of all of the observations recorded for each metric (Fig. 2B). Although there has long been a focus on estimating maximum performance, this is not a reliable metric for complex and voluntary behaviors (Dakin et al., 2020). Instead, a measure of the center captures the intrinsic differences between individuals, even if the sample sizes are small and if the animals do not achieve their true maximum performance during the sample period (Dakin et al., 2020). We used the median as the measure of center, because it is robust to potential outliers, which are likely due to artifacts in the sensor data (Segre et al., 2015).

To ensure that the sample median was an accurate estimate of an individual's performance, for each maneuver we only included whales that performed that maneuver more than 30 times. To arrive at this number, we conducted a simulation (Fig. S2A) with the assumption that the performance metrics were lognormally distributed. From a standard lognormal distribution representing a whale's ‘true’ performance capabilities, we randomly sampled with predefined sample sizes (range: 1 to 100) to represent the ‘observed’ performance, calculated the median and bootstrapped 95% confidence intervals. We performed 500 iterations for each predefined sample size, and then calculated the percentage of iterations where the ‘observed’ 95% confidence interval overlapped the ‘true’ median of the original lognormal distribution. We found that as sample size increased, the percentage of iterations where the ‘observed’ confidence intervals overlapped the ‘true’ median increased, stabilizing around 30 iterations (Fig. S2A).

We performed a similar analysis for the percentage of yaw-turns used out of the total number of turns used (YawTurn%), a metric of an individual whale's behavior. Because every turn is classified as either a pure-yaw turn or a banked turn, we repeated our subsampling analysis by randomly drawing from a Bernoulli distribution (with predefined sample sizes ranging from 1 to 100). Again, we found that beyond 30 observations, a high percentage of the ‘observed’ confidence intervals included the ‘true’ median of the original distribution.

Thus, for each maneuver we only included whales that performed that maneuver more than 30 times. However, after completing the scaling analysis described below, we reanalyzed the entire dataset using cut-offs of 50 and 100 observations per maneuver. Although this resulted in more individuals being excluded from the analysis, the general findings did not change.

### Repeatability of metrics

First, we needed to confirm that the performance metrics we measured were repeatable: an important requirement for being able to draw conclusions about how fixed traits (such as body size) affect highly variable traits (such as maneuvering performance; Dakin et al., 2020). If a performance metric is not repeatable across different days, then it cannot be used to describe an innate quality of an individual. To measure whether the performance metrics we selected were repeatable, we found 99 individuals in our dataset that had deployments spanning multiple days (range: 2–5 days). Of these whales, 20 had multiple tag deployments, including eight individuals where the deployments occurred in different years. For each calendar day, we calculated the median values for the six performance metrics and we calculated the YawTurn%. We only included daily medians that had >30 observations (Fig. S2), but we did not otherwise account for time of day or duration of deployment, both factors that would likely result in more repeatable median values if they were standardized. We then used a repeated-measures ANOVA (AnovaRM in the Python 3 ‘statsmodels’ package; Seabold and Perktold, 2010) with individual as the subject and day number as a within-subject factor to determine whether there were any significant differences between different days. We performed separate ANOVAs for whales that had deployments spanning two, three and four calendar days, and used a Bonferroni correction to account for multiple comparisons (α=0.002).

Another method of quantifying repeatability is the intra-class correlation coefficient (ICC), which is defined as the proportion of variation owing to differences among individuals (Nakagawa and Schielzeth, 2010; Segre et al., 2015). Owing to sample size considerations, we were only able to calculate the ICC for humpback whales and we were limited to only using two days per individual (*N*=39 to 44, depending on the metric).

### Body mass estimates

Body masses were estimated from body lengths (Table 1) using the species-specific equations found in Lockyer (1976). For each individual that was photographed by UAS, we estimated body mass using aerially measured body lengths (‘aerially measured whales’; *N*=93). For all individuals, we calculated body mass using species-average body length estimated from historical data (‘all whales’; *N*=280; data from Kahane-Rapport and Goldbogen, 2018; Lockyer, 1976).

### Scale dependent predictions of maneuvering performance

We used simple physics-based models to create predictions for how body mass (*m*) should affect maneuvering performance, with the assumption of geometric isometry. Under expectations of isometry, forward acceleration should scale with *m*^–1/3^ (Vogel, 2008). Starting with Newton's second law (Eqn 2), force scales with muscle cross-sectional area (*F*∼*l*^2^), and body mass scales with body length cubed (*m*∼*l*^3^):
(6)

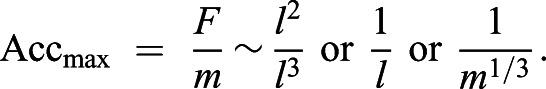


Centripetal acceleration (α_cent_) is the metric for pitch changes and turns (PitchD_cent_acc_, PitchU_cent_acc_, AllTurn_cent_acc_, YawTurn_cent_acc_), and under isometric assumptions it is predicted to scale with *m*^–1/3^. The derivation starts with Eqn 3, and assumes that all of the whales are swimming at the same translational velocity (*V*=constant across sizes), meaning that α_cent_ scales with the inverse of radius:
(7)

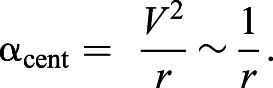


The direct relationship between the length of an arc and its radius (for a given subtended angle) suggests that the radius of a turn scales with body length (*r*∼*l*):
(8)

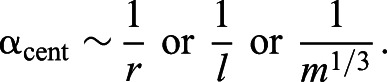


Rolling performance is predicted to scale with *m*^–2/3^. The derivation begins with Eqn 5, and then Eqn 4 is substituted for angular rolling acceleration (α_roll_):
(9)

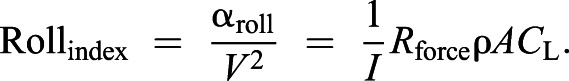


Density (ρ) and the coefficient of lift (*C*_L_) are constant across scale, leaving the scaling relationship:
(10)

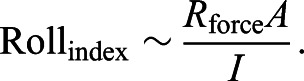


The radius at which the lifting force (a combination of flipper length and body radius) is applied scales with length (*R*_force_∼*l*), while the flipper area scales with length squared (*A*∼*l*^2^). Meanwhile, the moment of inertia (*I*) of a cylinder about its long axis is:
(11)

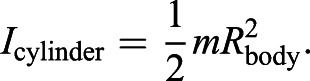


Body mass scales with body length cubed (*m*∼*l*^3^), while the radius of the body (*R*_body_) scales with length (*R*_body_∼*l*). Therefore, the moment of inertia scales with length to the fifth (*I*∼*l*^5^). Although ellipsoids and conical models of the whale body have different moments of inertia, the scaling relationship remains the same (Segre et al., 2016b). The end result is that Roll_index_ scales as follows:
(12)

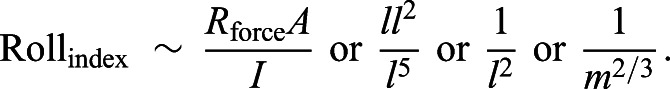


Finally, although we did not make specific predictions for how YawTurn% should scale with body mass, a reasonable null model is that it stays constant (*m*^0^).

### Statistical analysis

First, we calculated the effects of scaling on maneuvering performance using only the whales with aerial measurements of body size (Fig. 2C). We calculated the relationship between the log_10_ of the body mass and the log_10_ of each of the six performance metrics with a series of linear regressions (OLS in the Python 3 ‘statsmodels’ package; Seabold and Perktold, 2010). We originally used linear mixed-effects models (MixedLM in the ‘statsmodels’ package; with species as a random effect; Seabold and Perktold, 2010), but in all cases the variance owing to the species factor was low (with 95% confidence intervals overlapping 0). The results obtained from both statistical models were similar. Using ordinary least squares (OLS) regression, we calculated the slope and the 95% confidence intervals of the slope. If the slope predicted by the physics-based scaling models fell within the 95% confidence intervals of the regression line, we considered the differences to be non-significant. Next, we used a similar statistical analysis (OLS linear regressions after trying linear mixed models) to calculate the effects of scaling for all of the whales in the study, using the species-average mass derived from historical whaling data. We decided to perform the two analyses separately so that we would not mix and match estimates of body mass.

Generally, as lunge-feeding rorquals increase in body size, their dependence on krill increases (Nemoto, 1970). However, the relationship between prey type and body size in our dataset was more complex (krill feeders: minke, humpback, sei, fin, blue; fish feeders: Bryde's, humpback, fin; ghost shrimp: gray). To measure how behavior influences maneuvering performance, we focused on the humpback whale data because of the clear delineations in prey types. Out of the 131 humpback whales in the study, we had prey information for 123 individuals that were feeding. We used *t*-tests (Python3 ‘scipy’ package; Virtanen et al., 2020) to determine whether there was a difference in performance metrics between whales that were feeding on fish or on krill. We used Bonferroni corrections to account for multiple comparisons (α=0.007).

## RESULTS

We measured a total of 625,275 maneuvers from 280 individual whales (Table 1) over the course of 4037 deployment hours. There was a relatively even distribution of forward accelerations, pitch changes, turns and rolls (range: 109,050 rolls to 154,914 pitch-down rotations). Summary statistics for the seven performance metrics analyzed in this study are presented in Table 3. A total of 226,366 maneuvers were measured from 93 individual whales that were aerially photographed. Again, there was a relatively even distribution across the different types of maneuvers (range: 36,728 rolls to 58,133 pitch-down rotations).

**Table 3.**
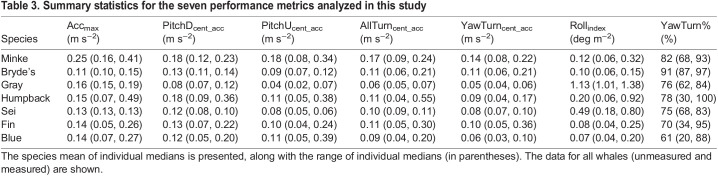
Summary statistics for the seven performance metrics analyzed in this study

### Repeatability of metrics

Our first measure of repeatability tested for differences in median performance between multiple calendar days (99 individuals). There was no significant difference for Acc_max_, PitchD_cent_acc_, PitchU_cent_acc_, Roll_index_ and YawTurn% (Table S2). There was a significant difference between days for AllTurn_cent_acc_ and YawTurn_cent_acc_, but only for whales that had deployments spanning 2 days.

Our second method of quantifying repeatability used the ICC to test for repeatability in humpback whales (39 to 44 individuals; using the first two calendar days of the deployment). For humpback whales, all seven performance metrics were considered repeatable (95% confidence intervals not overlapping 0), with two metrics (Acc_max_, PitchU_cent_acc_) classified as highly repeatable (>70%), two metrics (PitchD_cent_acc_, Roll_index_) classified as moderately repeatable (40–70%) and three metrics (YawTurn%, YawTurn_cent_acc_, AllTurn_cent_acc_) classified as having low repeatability (<40%; Segre et al., 2015).

### Scaling of maneuvering performance

**Fig. 3.**
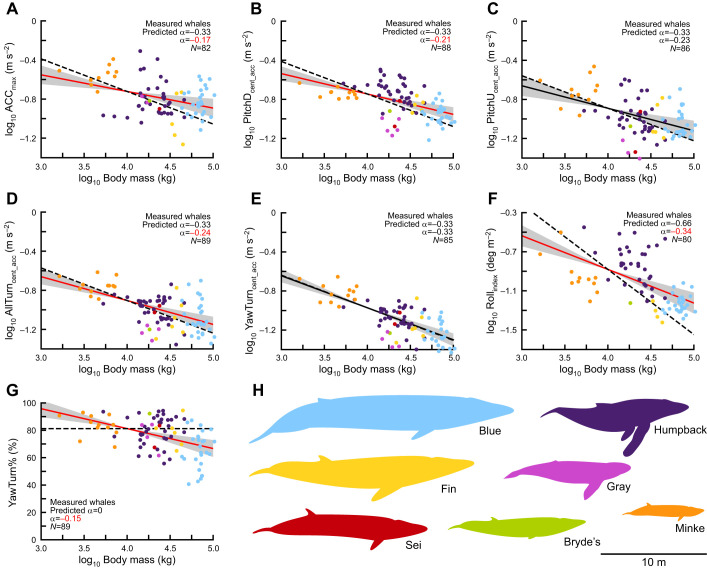
**Large whales exhibit positive allometry of maneuvering performance.** (A–G) The relationship between seven metrics of maneuvering performance and body mass (for aerially measured individuals) for seven species of whale. The predicted scaling relationships (dashed line), calculated scaling relationships (solid line; 95% CI of slope shown in gray), sample size and scaling coefficient (α) are shown (significantly different α are shown in red). Performance for forward accelerations (A), pitch-down rotations (B), turns (D) and rolls (F) exhibit positive allometry. Performance for pitch-up rotations (C) and yaw turns (E) are not significantly different than isometric predictions. When unmeasured whales are included in the analysis, similar results are obtained (Fig. 4). Percentage of yaw turns used (G) decreases with body mass. Species average body lengths are shown to scale (H). Owing to their unique feeding behaviors, sei and gray whales were not included in the roll analysis (see Fig. S4A for a version that includes these species).

**Fig. 4.**
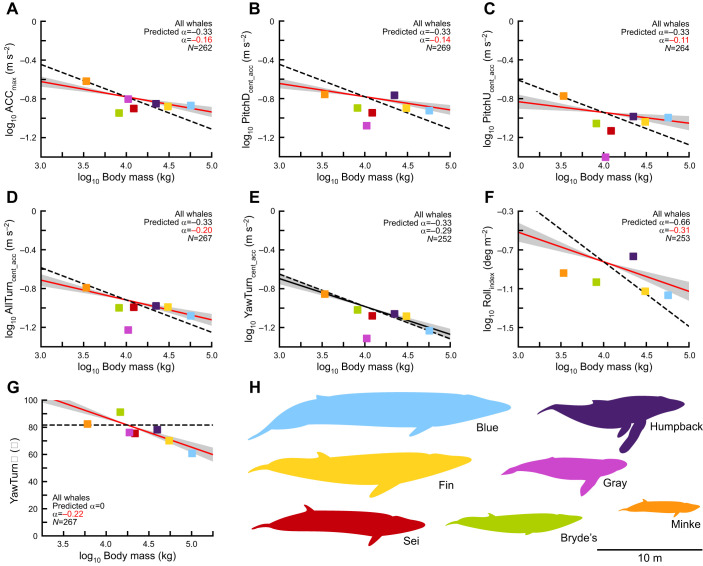
**When all whales (measured and unmeasured) are included in the analysis, the scaling patterns are similar to those obtained by examining aerially measured individuals (Fig. 3).** (A–G) The relationship between seven metrics of maneuvering performance and body mass for seven species of whale. The predicted scaling relationships (dashed line), calculated scaling relationships (solid line; 95% CI of slope shown in gray), sample size and scaling coefficient (α) are shown (significantly different α are shown in red). Performance for forward accelerations (A), pitch-down rotations (B), pitch-up rotations (C), turns (D) and rolls (F) exhibit positive allometry. Performance for pure-yaw turns (E) are not significantly different than isometric predictions. Percentage of yaw turns used (G) decreases with body mass. Species average body lengths are shown to scale (H). Owing to their unique feeding behaviors, sei and gray whales were not included in the roll analysis (see Fig. S4B for a version that includes these species).

Under expectations of isometry, forward acceleration (Acc_max_) was expected to scale with *m*^–1/3^ (scaling coefficient α=−0.33). For aerially measured whales, the scaling coefficient was significantly different than predicted (α=−0.17, *N*=82; Fig. 3A). For all of the whales in the study, the scaling coefficient was also significantly different than predicted (α=−0.16, *N*=262; Fig. 4A).

Centripetal acceleration was the metric for pitch changes and turns (PitchD_cent_acc_, PitchU_cent_acc_, AllTurn_cent_acc_, YawTurn_cent_acc_), and under isometric assumptions it was predicted to scale with *m*^–1/3^ (α=−0.33). For both aerially measured whales (α=−0.21, *N*=88; Fig. 3B) and all whales (α=−0.14, *N*=269; Fig. 4B), the scaling coefficient for PitchD_cent_acc_ was significantly different than predicted. For aerially measured whales, the scaling coefficient for PitchU_cent_acc_ was not significantly different than predicted (α=−0.23, *N*=86; Fig. 3C); however, for all of the whales in the study it was significantly different than predicted (α=−0.11, *N*=264; Fig. 4C). For AllTurn_cent_acc_, the scaling coefficients for both aerially measured whales (α=−0.24, *N*=89; Fig. 3D) and all whales (α=−0.20, *N*=267; Fig. 4D) were significantly different than the predicted values. Meanwhile, for both aerially measured whales (α=−0.33, *N*=85; Fig. 3E) and all of the whales (α=−0.29, *N*=252; Fig. 4E), the scaling coefficient for YawTurn_cent_acc_ was not significantly different from the predicted values.

Rolling performance was predicted to scale with *m*^–2/3^ (α=−0.66). For both aerially measured whales (α=−0.34, *N*=80; Fig. 3F) and all whales (α=−0.31, *N*=253; Fig. 4F), the scaling coefficient for Roll_index_ was significantly different than predicted. Sei whales and gray whales were not included in the analysis of rolls, as they had extremely high performance that was likely caused by their unique feeding behaviors. The proportion of pure-yaw turns used was predicted to stay constant across body sizes (α=0). However, for both aerially measured whales and all of the whales in the study, YawTurn% scaled negatively with body mass. For both aerially measured whales (α=−0.15, *N*=89; Fig. 3G) and for all of the whales in the study (α=−0.22, *N*=267; Fig. 4G), the scaling coefficients were significantly different than zero. The scaling coefficients and 95% confidence intervals for the scaling coefficients are shown in Figs 3 and 4.

### Effects of behavior on maneuvering performance

In humpback whales, behavioral traits affected individual maneuvering performance (Table 4). Compared with fish-feeders, krill-feeding humpback whales used higher performance accelerations (*P*<0.001) and pitch-up rotations (*P*<0.001). There was no significant difference between pitch-down rotations (*P*=0.011), turns (*P*=0.049), yaw turns (*P*=0.070) and proportion of turns used (*P=*0.730). Fish-feeding whales rolled faster than krill-feeding whales, but this difference was not significant when the Bonferroni correction was applied (*P*=0.009).

**Table 4.**
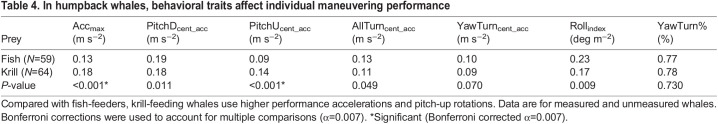
In humpback whales, behavioral traits affect individual maneuvering performance

## DISCUSSION

Maneuverability remains one of the most important and least understood aspects of locomotion. Animals rely on their maneuvering performance to catch prey, escape predators and defend territories (Altshuler, 2006; Walker et al., 2005; Wilson et al., 2015). Yet, because of the challenges involved with measuring maneuvering performance in the wild, little is known about this essential life function. In this study, we used bio-logging data, aerial photogrammetry and a high-throughput approach to study the maneuvering performance of free-swimming baleen whales, with the purpose of answering the question: how does maneuvering performance scale with body size in the world's largest swimming animals?

### Quantifying maneuverability in free-swimming whales

To quantify the maneuvering performance of baleen whales, we used a recently developed method that involves measuring the central tendencies of many voluntary maneuvers, performed across a range of behavioral circumstances (Dakin et al., 2018; Segre et al., 2015). The measure-of-center captures the intrinsic differences between individuals and is correlated with maximum performance, even if the animals do not achieve their true maximum performance during the sample period (Dakin et al., 2020). This method requires that the sample size for each individual is large enough (>30 per our analysis; Fig. S2), and that the central tendencies are repeatable (more variation between individuals than within individuals; Nakagawa and Schielzeth, 2010; Segre et al., 2015).

This study represents the first time that this approach has been applied to free-ranging animals. Previous experiments used this method to quantify the maneuvering performance of hummingbirds flying in a large cage (Dakin et al., 2018; Segre et al., 2015, 2016a). The experimental design of those studies was ideal for measuring repeatability (i.e. multiple trials conducted in similar environmental conditions over several weeks). However, those studies also found that not all maneuvers in the hummingbird repertoire were adequately sampled or repeatable in the artificial conditions provided (i.e. vertical flight performance; Segre et al., 2015). In this study we expected to find much higher variability within individuals, owing to the different deployment durations and the wide range of behavioral and motivational states that the whales could experience in the wild. Indeed, we did find high individual variation. Yet, we also found that for all the important categories of maneuvers, the median performance of individuals was not significantly different across calendar days (including for whales that were tagged multiple times in different months or years; Table S2).

We also attempted to replicate the analysis of repeatability used in the hummingbird studies by calculating the ICC. Owing to sample size constraints, this was only possible for humpback whales. Furthermore, because only 2 days were available for each individual, the confidence intervals were very large (Fig. S3). However, we found that none of the confidence intervals overlapped zero and therefore all these metrics were considered repeatable for individual humpback whales (Nakagawa and Schielzeth, 2010; Segre et al., 2015). Taken together, the results from these two analyses suggest that for all the maneuvers measured, individual performance was moderately repeatable across different days. Although this dataset is not ideal for quantifying repeatability (compared with carefully designed laboratory experiments), the finding that individual maneuvering performance was repeatable makes the analysis of scaling possible.

### Scaling of maneuvering performance

As body size increases, absolute maneuvering performance decreases (H1). Unsurprisingly, larger whales use lower forward accelerations and perform slower pitch-changes, turns and rolls than smaller whales. These patterns are evident in the graphs of the log-transformed data, where the slopes are all negative and the 95% confidence intervals of the slopes do not overlap with zero (Figs 3 and 4, solid lines).

However, baleen whales also exhibit positive allometry of maneuvering performance (H2). In other words, for most of our metrics, large whales outperformed our expectations. We expected forward acceleration to decrease proportionally with increasing body length (or *m*^–1/3^; Eqn 6), owing to the differential scaling of body mass and cross-sectional area of the locomotor muscles. Yet, we found that larger whales accelerate faster than isometric predictions. The reason why this occurs is not immediately clear, though it may reflect complex interactions between the allometric scaling of body proportions (Kahane-Rapport and Goldbogen, 2018), muscle size (Arthur et al., 2015), control surfaces (Kahane-Rapport and Goldbogen, 2018), hydrodynamic effects (Goldbogen et al., 2012; Gough et al., 2019) and the kinematics of specific maneuvers (Potvin et al., 2020).

Pitch-change and turning predictions were modeled after an arc that increases its length but preserves its central angle, and we expected these maneuvers to scale inversely with body length (or *m*^–1/3^). However, we found that large baleen whales outperform the expectations of scaling theory for pitch-changes and most turns. Cetaceans have high dorso-ventral flexibility, which they use to swim and to perform pitch-changes (Segre et al., 2019), and often generate lift with their extended flippers to increase centripetal acceleration (Segre et al., 2019). Spinal flexibility and lift generation may allow whales to perform pitch-changes faster than the isometric predictions based on shape suggest (Figs 3B,C and 4B,C). But with pure-yaw turns, whales cannot take advantage of their substantial dorso-ventral flexibility or lift from their flippers (Segre et al., 2019). In fact, pure-yaw turning performance matches well with isometric predictions, and the ability to flex laterally appears closely tied to body size (Figs 3E and 4E). However, we found that larger whales behaviorally compensate for their lack of lateral flexibility (H3). When pure-yaw and banked turns are considered together, baleen whales exhibit positive allometry for turning performance (Figs 3D and 4D). Larger whales use more banked turns than smaller whales (Figs 3G and 4G), which allows them to take advantage of their dorso-ventral flexibility and their lift-generating flippers to perform tighter turns (Segre et al., 2019). It is not clear why smaller whales do not use a similar strategy, although possible reasons may include energetic considerations, performance trade-offs, limits of body shape, or simply motivation. The end result is that larger whales have higher turning performance (but not higher yawing performance) than predicted by isometry.

We expected that rolling performance would scale with the inverse of length squared (or *m*^–2/3^), which would mean that larger whales have greatly reduced rolling ability. However, we found that rolling performance scales with approximately *m*^–1/3^ (Figs 3F and 4F). This suggests either that rolling performance exhibits strong positive allometry and that larger whales greatly outperform expectations, or that there may be a better model for the scaling of rolling performance that results in an isometric prediction of *m*^–1/3^. Compared with the other metrics that we measured, rolling performance has many interesting outliers. Unsurprisingly, humpback whales greatly outperformed the rolls of other species (Fig. 3F, and more clearly in Fig. 4F), and this is likely due to their long, scalloped flippers, which produce high levels of lift and torque (Fish and Battle, 1995; Miklosovic et al., 2004). However, gray whales and sei whales also had extremely high rolling performance, which was likely an artifact of unique behavioral states and low sample sizes. The gray whales were tagged while repeatedly suction feeding on the muddy bottom of shallow sounds. To perform this behavior, the whales swim at very low speeds while rolling to one side to touch their lips and baleen to the ground (Woodward and Winn, 2006). Aerial videos show that whales angle their flukes to the side and use them like rudders to perform the roll, with the flippers tucked against the body. This type of tail usage has been documented in other species of cetaceans, and probably can only be performed at slow speeds when the whale is not actively fluking (Fish, 2002, 2004). Importantly, tail ruddering represents a method of rolling that does not rely on forward speed to generate lift over the flippers, meaning that we would not expect these rolls to follow the flipper-generated lift model for performance. The two sei whales were also performing a unique surface feeding behavior where they maintained very slow translational speeds while lunging and rolling at high rates for several hours (Segre et al., 2021). For both gray whales and sei whales, the slow swimming speeds biased our rolling metric, which penalizes faster translational velocity (Eqn 5). Additionally, the repeated nature of these behaviors increased the individual median performance (see next section on how behavioral states influence measurement of performance). Although humpback whales are also known to maneuver at very slow speeds (Edel and Winn, 1978) and to use their flippers in non-traditional ways (Segre et al., 2017), the large number of individuals in our dataset, the longer deployments and the variety of behavioral states even out the median performance. For these reasons we did not include the gray and sei whales in the rolling analysis, even though including them does not substantially change the results (presented in Fig. S4).

**Fig. 5.**
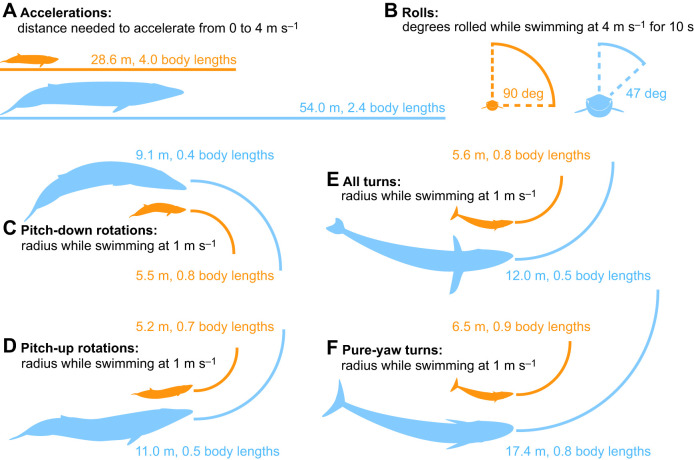
**Minke whales (orange) outmaneuver blue whales (blue) in absolute performance.** However, relative to their body size, blue whales outperform minke whales. Maneuvering performance is shown in easily interpretable and standardized conditions (i.e. turning radius at a given swimming speed). Performance measures shown are (A) accelerations, (B) rolls, (C) pitch-down rotations, (D) pitch-up rotations, (E) all turns and (F) pure-yaw turns.

Because the performance metrics that we selected are intuitively difficult to compare, we translated them into real-world values that are easier to interpret by standardizing swimming speeds, distances and time frames (Fig. 5). Our finding that performance decreases with body size is apparent in how blue whales require more distance to accelerate (Fig. 5A), roll across smaller angles (Fig. 5B) and perform wider radius turns (Fig. 5C–F) than the much smaller minke whales. However, our finding that larger whales outperform expectations can be seen in how blue whales need fewer body lengths to accelerate (Fig. 5A), and perform tighter turns relative to their body length (Fig. 5C–E) compared with minke whales. The exception is for pure-yaw turns: owing to their limited lateral flexibility, turning radius measured in body lengths is similar in blue and minke whales (Fig. 5F).

The scaling trends we observed are strongest in the aerially measured individuals, but similar trends are apparent when including non-aerially measured individuals (Fig. 4). We did not find significant scaling effects when looking within whale species. This is probably due to high variation in performance metrics, errors associated with body mass estimation, and a low range of body sizes (compared with across-species masses). In humpback whales, the most numerous species of our dataset, some of the same scaling trends are present but almost nothing is significant. By comparing the patterns observed in the measured whales (Fig. 3) and in all of the whales (Fig. 4) it becomes apparent that the scaling trends are driven by across-species body size, but are also affected by within-species body size.

### Effects of morphology on maneuvering performance

Larger whales are more maneuverable than predicted by body size alone, and this may be due to variation in the morphology of control surfaces. Different maneuvers are controlled with varying input from the flippers, flukes and the posture of the body (Segre et al., 2019). Meanwhile, morphological parameters such as fluke and flipper dimensions vary greatly within and across species. There are probably similar variations in dorso-ventral and lateral body flexibility, although this has not yet been measured. Within species, positive allometry of parameters such as flipper area may partially offset the decreased performance caused by larger body size (Kahane-Rapport and Goldbogen, 2018). Across species, drastic differences in the shape and size of control surfaces (Weber et al., 2014; Woodward et al., 2006) may account for some of the interspecific differences in performance (Figs 3 and 4). For example, high-aspect-ratio control surfaces generate more lift with less drag, producing more hydrodynamic force for maneuvering than low-aspect-ratio surfaces (Fish, 2004). Humpback whales appear to have enhanced rolling capabilities, and this may be a function of their long, high-aspect-ratio flippers (Fish and Battle, 1995). Gray whales, with their shorter and wider low-aspect-ratio flippers (Woodward et al., 2006), appear to have decreased ability to perform pitch-changes and turns, but this may also be a result of sampling bias. However, these differences remain anecdotal because we were not able to detect statistically significant species-level effects or within-species scaling trends. Morphology likely plays a complex and important role in influencing agility, but these effects appear to be swamped out by the inherent variation in our data.

### Effects of behavior on maneuvering performance

**Fig. 6.**
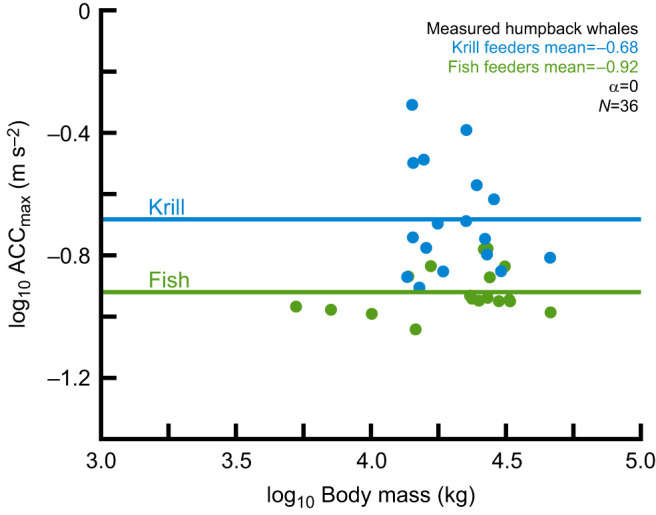
**In humpback whales, behavioral traits affect individual performance with fish-feeding individuals exhibiting a lower median acceleration than krill-feeding individuals (measured whales; neither slopes were significantly different than zero).** Compare with Fig. 3A.

Behavioral effects influence the median maneuvering performance of individuals, and this is an inescapable side effect of studying voluntary performance in the wild. There is an intrinsic stochasticity involved with measuring performance: is the individual sleeping or awake? Infirm or in top shape? Apathetic or highly motivated? Feeding or displaying to mates? Many baleen whales will only feed when aggregations of prey are sufficiently dense to yield a high net energy intake (Hazen et al., 2015). Capital breeding whales may spend months in a fasting state before traveling to a dedicated feeding ground, where they are observed feeding at high rates during short foraging seasons. For these whales, the timing when the tag deployment occurs likely influences the maneuvers recorded. Furthermore, prey density and type have a significant influence on the feeding style and predatory maneuvers used (Cade et al., 2016, 2020). Unsurprisingly, we found that even a coarse division of behavior (prey type pursued) influences median performance. Compared with fish feeders, krill-feeding humpback whales use higher performance accelerations and faster pitch-up rotations (Table 4). These differences in performance could simply reflect the types of maneuvers needed to catch different prey, rather than revealing intrinsic properties of the whales. Differences in behavior may underlie some of the variation that complicated the within-species scaling analysis (compare Fig. 6 with Fig. 3A). Future studies might find it beneficial to control for behavioral factors (e.g. prey type, feeding strategy, time of year), to reduce variability and better resolve the effects of scaling on within-species maneuvering performance.

Motivation and daily individual variation have always been one of the most difficult challenges involved with quantifying maneuvering performance. Studies that use highly motivational, natural events to measure maximal performance can be very informative but are not immune to this problem, as they rely on relatively low sample sizes of rare behaviors (Clark, 2009; Segre et al., 2020; Wilson et al., 2015). By sampling the same individual over a long period of time, our approach can capture a wider range of behavioral and motivational states, but is still limited in other ways. From the outset of this study, we expected there would be a high level of variability in the maneuvering data. We hoped (but were not confident) that the extended duration of some deployments, the high sample sizes of individuals and the large differences in body size would even out some of the variation. Ultimately, we found that the across-species trends in scaling are strong enough that they can be discerned, despite the natural variation and uncertainties associated with measuring voluntary behaviors in wild animals.

### Conclusions

One of the most fascinating characteristics of baleen whale biology is the ability of the world's largest animals to subsist entirely by chasing down and eating very small animals. Not only is this impressive from an energetic standpoint (Potvin et al., 2012, 2021), but it is also striking from a biomechanical perspective, as many small animals are inherently more agile than larger animals (Domenici, 2001; Fish, 2002). Both krill and bait fish have effective escape responses (Cade et al., 2020; Werth, 2012), which large cetaceans counter with a suite of morphological, kinematic and behavioral adaptations (Goldbogen et al., 2017a). Skim-feeding balaenids (right whales and bowhead whales) swim through patches of prey at speeds that are too slow to trigger escape responses (e.g. copepods; Werth, 2012). In comparison, lunge-feeding rorqual whales accelerate at high speeds through dense patches of krill or fish using energetically expensive, acrobatic maneuvers and carefully timed predatory strikes (Cade et al., 2020; Goldbogen et al., 2006, 2013; Segre et al., 2019). However, bait fish are more maneuverable than krill, and this may shape the ecological patterns of their predators (Domenici, 2001). As lunge-feeding rorquals increase in body size, their dependence on krill appears to increase; blue whales are both the largest species and obligate krill feeders, while smaller species can facultatively switch between prey types and often feed on fish (Nemoto, 1970). Indeed, our results suggest that larger whales suffer a dramatic reduction in maneuverability compared with their smaller counterparts (Fig. 5). However, we also found that larger whales are more agile than expected based on body size alone, and this may explain why some of the largest species (sei and fin whales) are known to occasionally feed on fish. But not all maneuvers are affected by body size in the same way, and larger whales choose maneuvers that they can perform effectively.

The ecology, evolution and behavior of baleen whales are shaped by many competing factors. Although gigantism allows for highly efficient prey capture (Goldbogen et al., 2019; Slater et al., 2017), it also has detrimental effects on the ability to outmaneuver prey. However, the positive allometry of maneuvering performance suggests that large whales have compensated for their increased body size by evolving more effective control surfaces and by preferentially selecting maneuvers that play to their strengths.

## Supplementary Material

Supplementary information
