# Supplementary material for: Scaling of maneuvering performance in baleen whales: larger whales outperform expectations
Source: J Exp Biol. 2022 Mar 2;225(5):jeb243224. doi: 10.1242/jeb.243224 (PMC8976943; doi:10.1242/jeb.243224)
Supplement: Supplementary information [file jexbio-225-243224-s1.pdf]

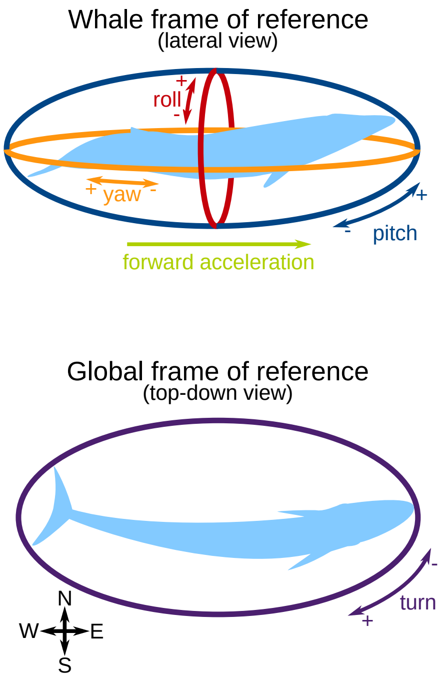

Fig. S1. Using the body orientation and speed data we identified six types of translational and rotational maneuvers. We measured forward accelerations, pitch-up and pitch-down rotations, rolls, and pure-yaw turns. We also measured turns that occurred in the global frame of reference, which included both pure-yaw turns and banked turns. When the whale is parallel to the surface, pitch is zero.

Journal of Experimental Biology • Supplementary information

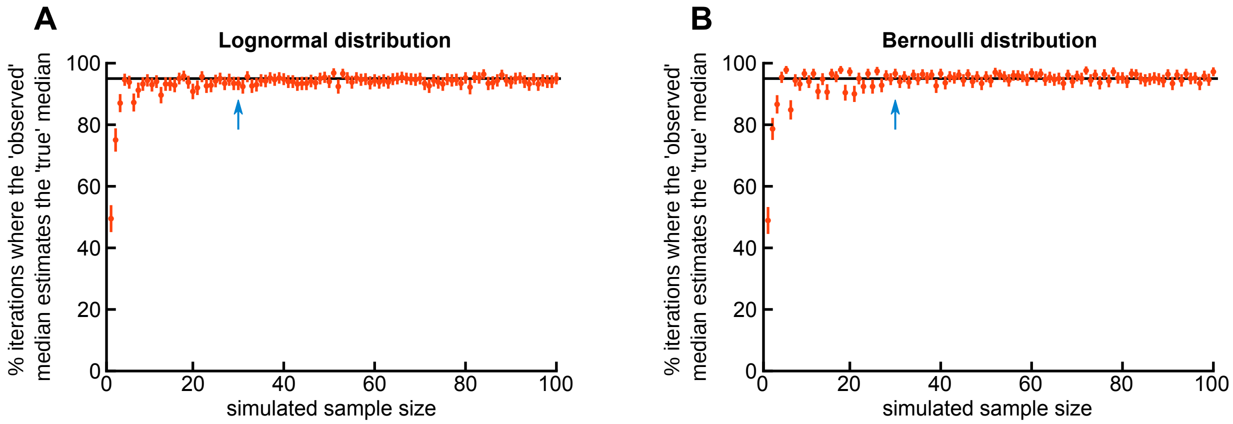

Fig. S2. For a given maneuver (accelerations, pitch-changes, turns), the minimum sample size needed to obtain an accurate measurement of the true median performance of an individual whale is 30 observations. (A) From a standard lognormal distribution representing a whale's 'true' performance capabilities (Figure 2B), we randomly sampled with predefined sample sizes (range: 1 to 100; x-axis) to represent the 'observed' performance, calculated the median, and bootstrapped 95% confidence intervals. We performed 500 iterations for each predefined sample size, and then calculated the percentage of iterations where the 'observed' 95% confidence interval overlapped the 'true' median of the original lognormal distribution (y-axis). The error associated with these simulations are shown as 95% confidence intervals (red vertical lines). We found that as sample size increased, the percentage of iterations where the 'observed' confidence intervals overlapped the 'true' median increased, stabilizing around 30 iterations (blue arrow). (B) We performed a similar analysis for the percentage of yaw-turns used out of the total number of turns used (YawTurn%). Since every turn is classified as either a pure-yaw turn or a banked turn, we repeated our subsampling analysis by randomly drawing from a Bernoulli distribution (with predefined sample sizes ranging from 1 to 100; x-axis). Again, we found that beyond 30 observations (blue arrow), a high percentage of the 'observed' confidence intervals included the 'true' YawTurn% from the original distribution.

Journal of Experimental Biology • Supplementary information

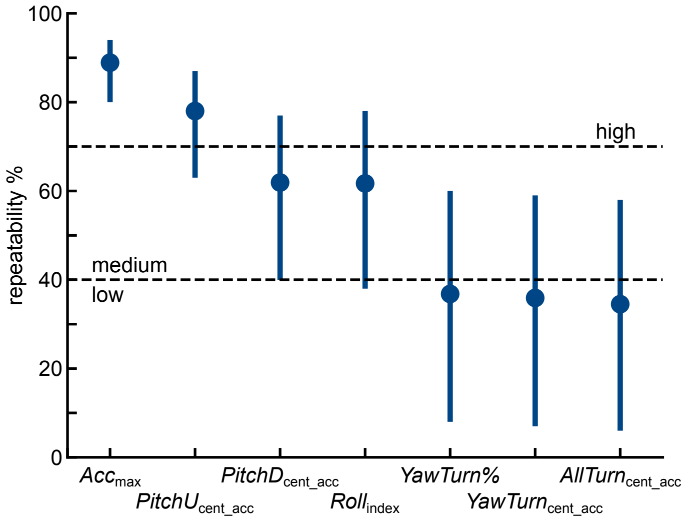

Fig. S3. Another method of quantifying repeatability is the intra-class correlation coefficient (ICC), which is defined as the proportion of variation due to differences among individuals (Nakagawa and Schielzeth, 2010; Segre et al., 2015). Due to sample size considerations, we were only able to calculate the ICC for humpback whales and we were limited to only using two days per individual (N =39 to 44). For humpback whales, all seven performance metrics are considered repeatable (95% confidence intervals not overlapping 0), with two metrics (Accmax, PitchUcent_acc) classified as highly repeatable, two metrics (PitchDcent_acc, Rollindex) classified as moderately repeatable, and three metrics (YawTurn%, YawTurncent_acc, AllTurncent_acc) classified as having low repeatability (Segre et al., 2015).

Journal of Experimental Biology • Supplementary information

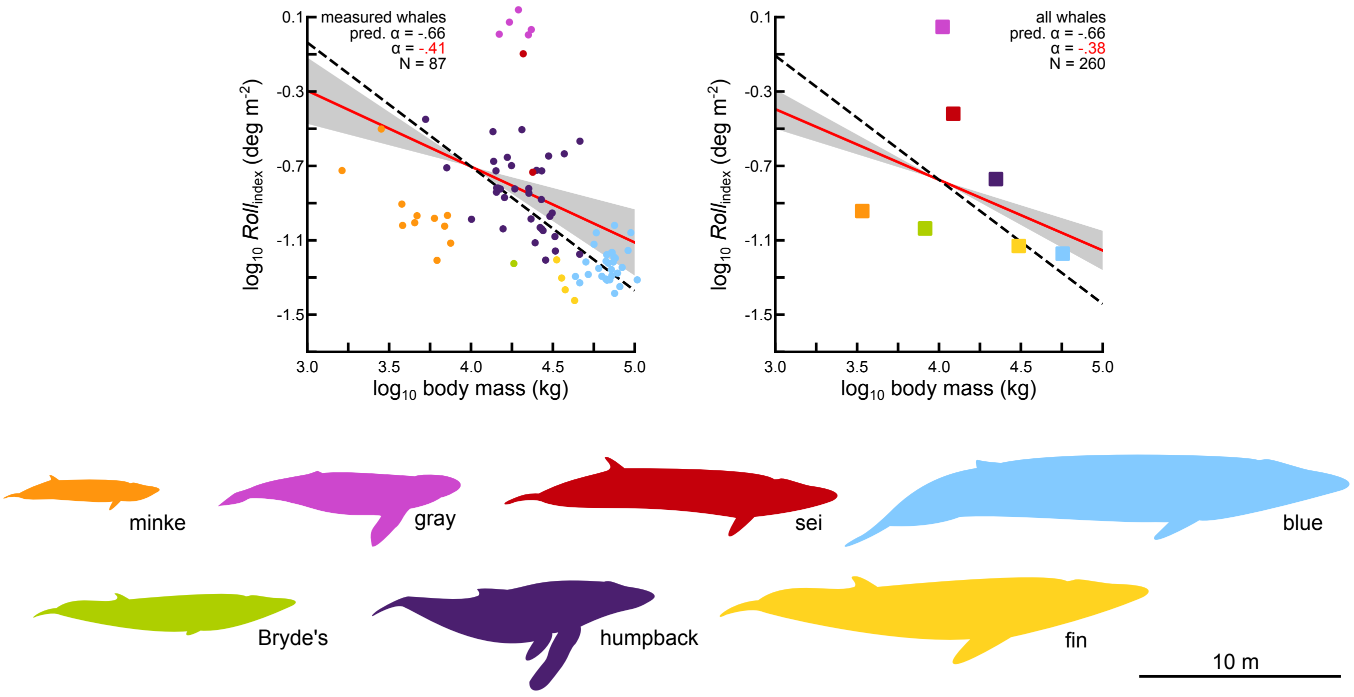

Fig. S4. When sei whales and gray whales are included in the analysis of rolling performance, the overall conclusions do not change. Due to the behavioral states of the tagged individuals, the sei and gray whales that we measured have very high median rolling performance. However, including them in the scaling analysis for rolling maneuvers (Figure 3F, Figure 4F) does not change the conclusions that large whales outperform expectations.

Table S1. A list of Unoccupied Aircraft Systems (UASs) used to photograph and measure whales.

species	location	UAS	altimeter	notes	references
minke	Antarctica	LemHex-44	laser	1	*
		FreeFly Alta 6	laser	1	*
Bryde's	South Africa	Dji Phantom 4 Advanced	barometric	2, 3	*
gray	Puget Sound	Aerial Imaging Solutions APO-42	laser	4	**
humpback	Alaska	DJI Inspire 2	barometric	5	
	Antarctica	LemHex-44	laser	1	*
		FreeFly Alta 6	laser	1	*
	Greenland	DJI Phantom 4	barometric	2, 3	*
	California	LemHex-44	laser	1	*
		FreeFly Alta 6	laser	1	*
		DJI Phantom 4A	barometric	2, 3	*
sei	Falklands	DJI Phantom 4A	barometric	2, 3	*
fin	Azores	DJI Phantom 4p	laser	6	*,***
	Greenland	DJI Phantom 4A	barometric	2, 3	*
blue	California	LemHex-44	laser	1	*
		FreeFly Alta 6	laser	1	*
		DJI Phantom 4A	barometric	2, 3	*

1) Sony Alpha A5100, Sony SEL50 50 mm or SEL35 35 mm focal length low distortion lens
2) DJI integrated camera
3) scale confirmed with floating calibration object
4) Sony A7R, Sony Sonar T FE 55mm lens
5) DJI Zenmuse X75S, 25mm Olympus lens
6) Custom-built laser altimeter added

* Gough et al 2019
** Durban et al 2015, Durban et al 2016

*** Dawson 2017, for a description of a customized laser altimeter

Journal of Experimental Biology • Supplementary information

Table S2. To quantify repeatability of maneuvering performance metrics within individuals, we took the deployments that spanned multiple calendar days and the individuals that had multiple deployments across different days, months, or years (N = 20) and calculated the daily median for each metric. We only used daily medians that had >30 observations (Figure S2). We then used a repeated-measures ANOVA to determine if there were any significant differences between different days. We performed separate analyses for whales that had deployments spanning two, three, and four calendar days, and used a Bonferroni correction to account for multiple comparisons (α = 0.002). These results suggest that within individuals, performance is repeatable across days.

# days	# whales	Acc_{max}	$PitchD_{cent\_acc}$	$PitchU_{cent\_acc}$	$AllTurn_{cent\_acc}$	$YawTurn_{cent\_acc}$	$Roll_{index}$	*YawTurn%*
2	62 - 74	0.30	0.90	0.02	**0.001***	**< 0.001***	0.42	0.02
3	8 - 11	0.73	0.24	0.29	0.11	0.046	0.95	0.45
4	4 - 6	0.57	0.97	0.22	0.76	0.47	0.27	0.90

*significant (Bonferroni corrected α = 0.002)

Journal of Experimental Biology • Supplementary information

Supplementary Materials and Methods

Derivation of Equation 4

adapted from *Segre et al. 2016*

To perform a roll, a rorqual whale extends its flippers and generates an upwards lifting force with one flipper and a downwards lifting force with the other flipper:

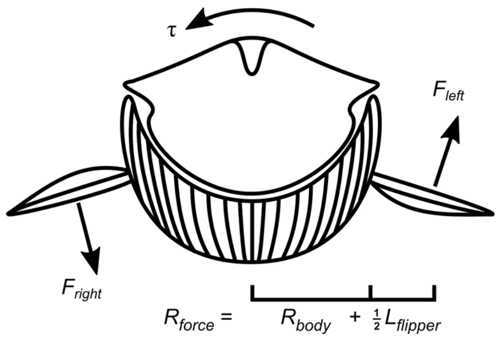

(adapted from *Segre et al 2016*)

The hydrodynamic force (F in Newtons) from each flipper is calculated with the lift equation:

$$F_{left\ or\ right} = \frac{1}{2}\rho A C_L V^2$$

(eq S1)

where ρ is the density of salt water, A is the area of the flipper (m^2), C_L is the non-dimensional coefficient of lift, and V is the translational velocity (or swimming speed, in m s^{-1}). The force from each flipper is applied at radius (R_{force}) which is the sum of the radius of the body (R_{body}) and half the length of the flipper ($L_{flipper}$), to produce a net torque (τ in N m):

$$\tau = R_{force} F_{left} + R_{force} F_{right}$$

(eq S2)

The torque produces an angular acceleration (α_{roll} in rad s^{-2}), which is calculated using the rotational analog of Newton's second law:

$$\alpha_{roll} = \frac{\tau}{I}$$

(eq S3)

where I is moment of inertia of the whale about the long axis (kg m^2). Combining equations S1-S3 yields equation 4:

$$\alpha_{roll} = \frac{1}{I} R_{force} \rho A C_L V^2$$

(eq 4)

Journal of Experimental Biology • Supplementary information

Supplementary References

Dawson, S. M., Bowman, M. H., Leunissen, E., and Sirguey, P. (2017) **Inexpensive aerial photogrammetry for studies of whales and large marine animals.** *Front. Mar. Sci. 4, 366.*

Durban, J. W., Fearnbach, H., Barrett-Lennard, L. G., Perryman, W. L. and Leroi, D. J. (2015). **Photogrammetry of killer whales using a small hexacopter launched at sea.** *J. Unmanned Veh. Syst.* **3**, 131–135.

Durban, J. W., Moore, M. J., Chiang, G., Hickmott, L. S., Bocconcelli, A., Howes, G., Bahamonde, P. A., Perryman, W. L. and LeRoi, D. J. (2016). **Photogrammetry of blue whales with an unmanned hexacopter.** *Mar. Mamm. Sci.* **32**, 1510–1515.

Gough, W. T., Segre, P. S., Bierlich, K. C., Cade, D. E., Potvin, J., Fish, F. E., Dale, J., di Clemente, J., Friedlaender, A. S., Johnston, D. W., et al. (2019). **Scaling of swimming performance in baleen whales.** *J. Exp. Biol.* **222**, jeb204172.

Segre, P. S., Cade, D. E., Fish, F. E., Potvin, J., Allen, A. N., Calambokidis, J., Friedlaender, A. S. and Goldbogen, J. A. (2016). **Hydrodynamic properties of fin whale flippers predict maximum rolling performance.** *J. Exp. Biol.* **219**, 3315-3320.
